# Editorial: Towards 2030: sustainable development goal 1: no poverty. A sociological perspective

**DOI:** 10.3389/fsoc.2024.1487228

**Published:** 2024-09-17

**Authors:** Andrzej Klimczuk, Grzegorz Piotr Gawron, Piotr Toczyski

**Affiliations:** ^1^Department of Social Policy, Collegium of Socio-Economics, SGH Warsaw School of Economics, Warsaw, Poland; ^2^Institute of Sociology, Faculty of Social Sciences, University of Silesia in Katowice, Katowice, Poland; ^3^Media and Communication Department, Institute of Philosophy and Sociology, The Maria Grzegorzewska University, Warsaw, Poland

**Keywords:** SDG1, COVID-19, poverty, injustice, resilience, sustainable development

## Overview

This Research Topic focuses on the first Sustainable Development Goal (SDG) by the UN, which aims to “end poverty in all its forms everywhere.” Progress toward this goal is evaluated through various targets and indicators. The most recent SDG progress report highlighted how poverty reduction has significantly slowed and worsened, mainly due to the COVID-19 pandemic (United Nations, 2024). This unprecedented emergency has particularly affected informal workers, young people, and women. With the goal of eradicating poverty by 2030 currently off track, intensified action is crucial. Moreover, amid a growing climate crisis, significant efforts are needed to bring the 2030 targets within reach. This Research Topic examines SDG1 from a sociological perspective, focusing on its framing, geographic adaptations, stakeholder involvement, and the influence of social mobility and stratification studies, especially in the context of the challenges of the post-COVID-19 pandemic.

Two journals were involved in the work on this project: “Frontiers in Sociology” and “Frontiers in Human Dynamics.” The presented Research Topic includes four articles prepared by six authors affiliated from the following countries: Denmark, Italy, Türkiye, and the United Kingdom. Three types of articles are included, covering: two original research articles (Bova; Millard and Fucci), one review (Özgün and Dolcerocca), and one mini review (Hung). This Research Topic covers topics encompassing, among others, definitions and measurement of poverty and social exclusion, the COVID-19 pandemic emergency measures, health policies, mental health, homelessness, internal migration, social reintegration, resilience, agency and structure, and social innovation.

## Selected studies

In the first study included in this Research Topic, “*Bringing classes back into poverty discussions*,” Özgün and Dolcerocca focused on a shift in the poverty discourse that started in the 1980's and on moving from production relations to consumption relations, using measurements of consumption levels and purchasing power to define poverty. This approach often relies on absolute poverty lines or relative indicators to differentiate between “poor” and “non-poor” populations. The paper argues that, while these assessments can track trends over time, they hide the root causes of poverty and lead to overly optimistic interpretations. The study critiques the decontextualization of poverty from its political and economic context, particularly within a neoliberal framework. Moreover, this research advocates for a focus on the material causes of poverty, which are rooted in the conflicts inherent in the capitalist system and the class-based contradictions of production.

The research team of Millard and Fucci continued the discussion on fundamental issues in their research on “*The role of social innovation in tackling global poverty and vulnerability*.” The authors underlined recent events and crises, such as the financial crisis of 2007–2008, the COVID-19 pandemic, and the war in Ukraine, which have contributed to the rapid rise in global poverty. The study argues that development organizations have long used social innovation to combat poverty, but only recently have they begun to systematically analyze its contributions to the SDGs. Social innovation, driven by local civil society involvement, aims to empower those in need by increasing their agency and capability rather than relying on external aid. This study examines various definitions of poverty and advocates for recalibrating social innovation to address contemporary challenges, emphasizing the need for proactive public sector involvement. Furthermore, the article calls for a revised understanding of the relationship between sustainable development and resilience, proposing a nexus approach that integrates SDG1 with other related goals.

Two studies focus on case studies related to poverty. In the paper, “*The homeless population during the COVID-19 syndemic: inequities, practices of social resilience, and social reintegration strategies*,” Bova examined the social insecurity and misrecognition of people experiencing homelessness during the COVID-19 pandemic in Bergamo, Italy. The research was conducted in two phases. The first focused on analyzing the social resilience practices of socio-educational staff and support services during the crisis. During the second research phase, social dynamics that can improve the long-term wellbeing and reintegration of homeless people have been studied. Initially, public authorities neglected people without housing, prompting social services to implement emergency responses and acquire protective equipment. The article highlights that to achieve full social reintegration and prevent future misrecognition, fostering a relationship with a non-stigmatized social community and involving the local community in awareness-raising and participatory activities are essential for developing support networks.

In the final paper, “*Is psychologically vulnerable rural-to-urban migrants' mental health further at stake under China's tightened COVID-19 measures: how should the government respond?*” Hung studied potential policy recommendations for the recent population changes. The study indicates that the COVID-19 outbreak has had a widespread effect on the mental health of Chinese nationals, but its impact on rural-to-urban migrants has not been well-documented. The author argues that rural-to-urban migrant workers already face discrimination and stigma in urban areas, which severely influences their mental health. The pandemic is likely to have exacerbated these issues, although this has been insufficiently explored. To address these challenges, Chinese policymakers need to understand the specific mental health impacts on these migrants to create effective interventions. The researcher calls for local governments to adopt policies to mitigate the mental health burden of rural-to-urban migrants, given their specific characteristics and social risks.

## Conclusion

The research findings contained in the papers in this Research Topic allow the formulation of at least four directions for further research. These are (1) new approaches to defining and measuring poverty in the context of other SDG agendas, such as SDG3 related to public health challenges (Atkinson, 2019); (2) scaling up of social innovations related to poverty reduction (Nicholls and Ziegler, 2019); (3) fostering the institutional ecosystems to support the social economy and solidarity economy (Fontan and Lévesque, 2023); and (4) comparative social policy studies, including differences between countries and a variety of policy ideas and policy instruments (Aspalter, 2021).
